# A Review of New-Onset Ventricular Arrhythmia after Left Ventricular Assist Device Implantation

**DOI:** 10.1159/000524779

**Published:** 2022-04-28

**Authors:** Jianwei Shi, Xinyi Yu, Zhigang Liu

**Affiliations:** Department of Cardiovascular Surgery, TEDA International Cardiovascular Hospital, Chinese Academy of Medical Sciences & Peking Union Medical College, Tianjin, China

**Keywords:** Left ventricular assist device, Ventricular arrhythmia

## Abstract

**Introduction:**

Heart failure (HF) is a severe and terminal stage of various heart diseases. Left ventricular assist devices (LVADs) are relatively mature and have contributed to the treatment of end-stage HF. Ventricular arrhythmia (VA) is a common complication after LVAD implantation, including ventricular tachycardia and ventricular fibrillation, both of which may cause abnormal circulation.

**Methods:**

A literature search was conducted in the PubMed database, “Ventricular Arrhythmia” OR “VA” OR “Arrhythmia” OR “Ventricular Tachycardia,” OR “Ventricular Fibrillation” AND “LVAD” OR “Left Ventricular Assist Device” OR “Heart Assist Device” as either keywords or MeSH terms, the authors screened the titles and abstracts of the articles. Eventually, 12 original research articles were retrieved.

**Results:**

The 0.83 [95% CI: 0.77, 0.89] of patients were male. A whole of 53% [95% CI: 0.25, 0.81] of VA patients had a history of atrial fibrillation and 61% [95% CI: 0.52, 0.69] had a history of VA. 39% [95% CI: 0.29, 0.49] of the participants had no prior history of VA and experienced new VA following CF-LVAD implantation. Following CF-LVAD implantation, 59% [95% CI: 0.51, 0.67] of patients developed early VA (VA ≤30 days). The 30-day mortality rate of patients was 4% [95% CI: 0.01, 0.07]. And overall mortality was 28% [95% CI: 0.15, 0.41]. The reported incidence of VA after LVAD implantation is not identical in different medical centers and ranges from 20% to 60%. The mechanism of VA after LVAD implantation is summarized as primary cardiomyopathy-related, device mechanical stimulation, myocardial scarring, ventricular displacement, electrolyte regulation, and other processes.

**Conclusions:**

A preoperative VA history is considered a predictor of VA following LVAD implantation in most studies. Multiple mechanisms and factors, such as prevention of “suction events,” ablation, and implantable cardioverter defibrillator, should be considered for the prevention and treatment of postoperative VA in patients requiring long-term VAD treatment. This study provides a reference for the clinical application of LAVD and the prevention of postoperative VA after LVAD implantation. Future multicenter prospective studies with uniform patient follow-up are needed to screen for additional potential risk factors and predictors. These studies will help to define the incidence rate of VA after LAVD implantation. As a result, we provide guidance for the selection of preventive intervention.

## Introduction

Heart failure (HF) is a severe and terminal stage of various heart diseases. The prevalence of HF is steadily increasing. More importantly, HF is a disease that consumes significant health care resources, causes noteworthy morbidity and death, and has an important effect on the quality of life [1]. HF is a global health care epidemic that affects approximately 26 million people globally and causes over 1 million hospitalizations in both the USA and Europe each year [2]. Despite significant advances in the pharmacological treatment of HF, heart transplantation remains the optimal treatment for patients with severe or end-stage HF. However, the use of heart transplantation is limited due to factors such as a lack of donors.

Therefore, mechanical circulatory assist devices, as represented by ventricular assist devices (VADs), have gradually become the method of choice during the transitional period of heart transplantation and even an alternative to the long-term treatment of end-stage HF [3]. Among these devices, the left ventricular assist device (LVAD) is relatively mature and has contributed to the treatment of end-stage HF. The incidence of ventricular arrhythmia (VA) after LVAD implantation ranges from 20% to 60% [4]. Ventricular tachycardia (VT) and ventricular fibrillation (VF), both of which may cause loss of perfusion of systemic circulation and pulmonary circulation, low cardiac output, and suspended vital tissue and organ perfusion [5]. Although clinical symptoms of LVAD-assisted patients may be indistinct in a short time, persistent VA may lead to right heart dysfunction, which affects circulating flow and eventually leads to hemodynamic compromise [6, 7, 8]. We aimed to summarize the characteristics of VA after LAVD implantation, perioperative management strategies, and guidance regarding methods for out-of-hospital prevention by reviewing recent case reports, epidemiological studies, risk factors, and prevention and treatment strategies for VA after LVAD implantation and to provide a reference for the clinical application of LAVD and the prevention of postoperative VA after LVAD implantation.

## Materials and Methods

### Literature Search Strategy

Complete electronic searches were performed in September 2021 by using PubMed. To achieve the maximum sensitiveness of the search strategy, combined terms were used: “Ventricular Arrhythmia” OR “VA” OR “Arrhythmia” OR “Ventricular Tachycardia,” OR “Ventricular Fibrillation” AND “LVAD” OR “Left Ventricular Assist Device” OR “Heart Assist Device” as either keywords or MeSH terms in PubMed. All qualifying references were combed through for additional possibly relevant research, which was then appraised using the inclusion and exclusion criteria.

### Selection Criteria

All English-language publications with data on patients over the age of 18 who developed VA after LVAD implantation were examined for inclusion. Articles were omitted if they did not include the information about patients who developed VA after receiving LVAD. Only the most complete reports with the longest follow-up term were selected for quantitative assessment when institutions published duplicate studies with overlapping data on patients and follow-up periods. Studies that were not published in English and did not include human participants were omitted. Also omitted were abstracts, case reports, conference presentations, editorials, reviews, and expert opinions.

### Definition of VA

VA was defined as sustained (>30 s) VT or any episode of VF. In this study, we only focused on patients who developed VA after CF-LVAD implantation.

### Data Extraction and Critical Appraisal

Two reviewers collected data from article texts, tables, and figures. Discussion and consensus were used to address inconsistencies between the two reviewers.

### Statistical Analysis

All analyses were performed with the Review Manager (RevMan) (computer program). Version 5.4 (The Cochrane Collaboration, 2020). Heterogeneity was evaluated using the *I*^2^ test. *p* values <0.05 were considered statistically significant.

## Results

### Study Characteristics

**Fig. 1:**
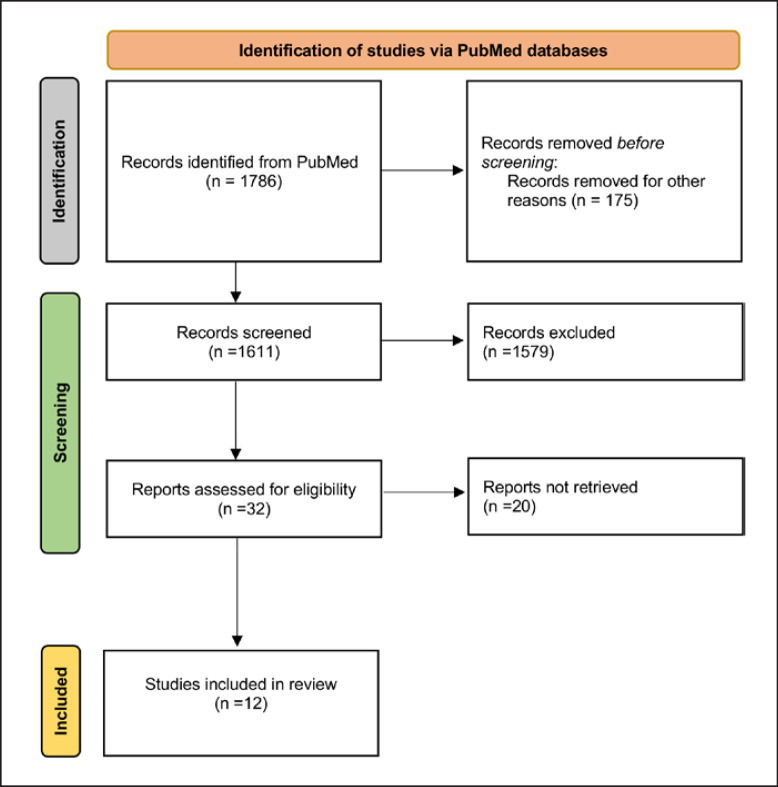
PRISMA schematic of the search strategy. PRISMA, Preferred Reporting Items for Systematic Reviews and Meta-Analyses.

In all, 1,786 records published were found in the literature, and eventually, 12 studies were included in the analysis after applying the inclusion and exclusion criteria. All of the studies were retrospective or prospective studies that investigated VA occurred after LVAD implantation. A manual search of references did not yield further studies. A PRISMA flow diagram displaying the entire search strategy is shown in Figure 1, and a thorough overview of the papers used for analysis is provided in Table 1. This research included a total of 456 individuals who suffered VA after receiving LVAD.

### Baseline Demographics

Baseline demographics of patients who developed VA are shown in Table 2. The 0.83 [95% CI: 0.77, 0.89] of patients were male. A total of 0.40 [95% CI: 0.32, 0.49] of patients had diabetes mellitus history and 0.58 [95% CI: 0.52, 0.64] had hypertension history. HeartMate II LVAD was used by 0.85 [95% CI: 0.78, 0.93] patients, whereas HeartWare HVAD was used by just 0.04 [95% CI: 0.01, 0.06]. 3% [95% CI: 0.00, 0.05] of patients used the other type of device model. In 58% [95% CI: 0.45, 0.70] of patients, the LVAD was used as a bridge to transplantation, and in 42% [95% CI: 0.29, 0.54] of cases, it was used as destination therapy.

### Characteristics of VA

**Fig. 2:**
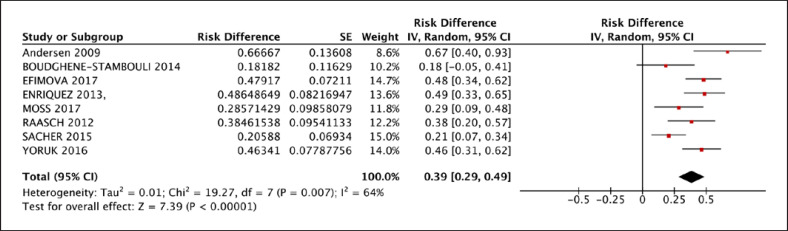
New VA rate reported for patients who experienced VA after LVAD implantation.

**Fig. 3:**
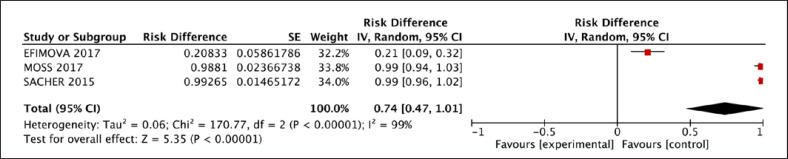
Ablation rate reported in patients for treatment of VA after LVAD implantation.

A total of 34% [95% CI: 0.28, 0.40] of patients who received LVAD had VA during LVAD therapy. A whole of 53% [95% CI: 0.25, 0.81] of VA patients had a history of atrial fibrillation and 61% [95% CI: 0.52, 0.69] had a history of VA, which is shown in Table 3. Thirty-nine percent [95% CI: 0.29, 0.49] of the participants had no prior history of VA and experienced new VA following CF-LVAD implantation (Fig. 2). Following CF-LVAD implantation, 59% [95% CI: 0.51, 0.67] of patients developed early VA (VA ≤30 days). For treatment of VA, anti-tachycardiac pacing (ATP) was given to 0.27% [95% CI: 0.04, 0.49], cardioversion was given in 69% [95% CI: 0.57, 0.82], and ablation was performed in 74% [95% CI: 0.47, 1.01] of patients (Fig. 3).

### Outcomes of Patients with Post-LVAD VA

**Fig. 4:**
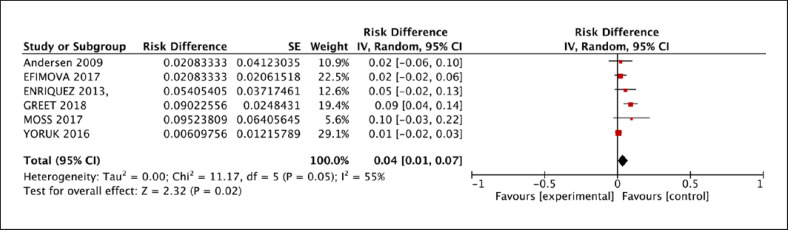
Early (≤30 days) mortality rate reported for patients who experienced VA after LVAD implantation.

**Fig. 5:**
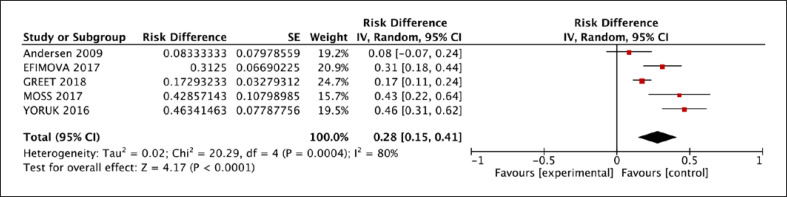
Overall mortality rate reported for patients who experienced VA after LVAD implantation.

The 30-day mortality of patients was 4% [95% CI: 0.01, 0.07] (Fig. 4), and overall mortality was 28% [95% CI: 0.15, 0.41]. 25% [95% CI: 0.13, 0.36] of VA patients after LVAD implantation were transplanted eventually (Fig. 5). Postoperative outcomes are outlined in Table 4.

## Case Reports and Epidemiological Studies

VA is more common within the first 30 days after LVAD implantation, and approximately one-third of patients may progress to terminal VA [9, 10, 11]. LVADs do not rely on heart rate to sustain cardiac output directly, and thus symptoms are frequently milder and hemodynamics are more stable in LVAD-assisted patients with short-term VA [4]. A case of VA with HeartWare® was reported by Smith and Moak [12]. A 50-year-old female patient was sent to the hospital with a low flow alert on the device and periodic dizziness 6 years after LAVD implantation. A physical examination revealed VF, which was treated with electrical cardioversion. Other similar reports [13, 14, 15] also documented that the patients' circulation was relatively stable and clinical symptoms were modest. Long-term VA persistence or recurrence following LVAD implantation is linked to hemodynamic failure [11]. Multiple recurrences of VF and eventually secondary multiorgan failure due to right ventricular system failure were reported by Jakstaite et al. [16] in a 61-year-old male patient who had been using a continuous-flow LVAD (CF-LAVD) for 7 years.

Because of the different types of LVADs used, multiple study populations, follow-up times, and various etiologies of HF in patients, as well as the differences between results from single-center and multicenter trials, the reported incidence of VA after LVAD implantation is not identical in different medical centers. Harding et al. [17] and Andersen et al. [18] conducted single-center investigations with sample sizes of 17 and 23 patients, respectively, and observed a 59% and 52% incidence of postoperative VA, respectively. The difference between the two studies is the type of LVAD implanted, which was a pulsatile flow pump in the study by Harding and a continuous flow pump in the study by Andersen. Garan et al. [19] studied clinical data from 162 LVAD-assisted patients and discovered a 23.5% incidence of early postoperative VA (within 30 days of surgery). Cantillon et al. [20] reviewed the clinical data from 478 patients who underwent LVAD implantations at Cleveland Medical Center between 1991 and 2008 and found that the incidence of postoperative VA was 28.9%. Yap et al. [7] focused on 204 patients with LVAD implantations at two medical facilities in the Netherlands and found that 30.4% of them experienced postoperative VA. Miller et al. [10] conducted a multicenter study with 133 patients who had a continuous flow pump implanted and found that 24% of the patients developed postoperative VA. Bedi et al. [21] evaluated the clinical data from 111 patients who underwent LVAD implantations and discovered that 22% of them had postoperative VA.

Bedi et al. [21] discovered a 54% mortality risk for the development of VA within 1 week after LVAD implantation; patients with VA during LVAD support had a considerably higher mortality rate (33% vs. 18%, *p* < 0.001) than patients without VA [21]. Brenyo et al. [22] retrospectively reviewed clinical data from 61 patients with LVADs and reported that VA that developed within 1 month of LVAD implantation was related to a 45% mortality rate, and multivariate analysis revealed that postoperative VA was a predictor of higher mortality risk (HR = 9.69, *p* = 0.001). Galand et al. [43] showed that early VA after LVAD implantation was the greatest predictor of postoperative death within 30 days after the operation (OR = 7.36, *p* = 0.001).

## Discussion

Overall, we discovered numerous significant tendencies in the process of VA after patients received LVAD. About 60% of patients who developed VA after LVAD implantation had a prior VA history, while nearly 40% had a new-onset VA. The majority of patients who occurred VA within the first 4 weeks of CF-LVAD implantation, and around half of them had symptomatic VA. When compared to historical controls, our research found that short-term mortality in patients with VA after LVAD implantation was equivalent to that of patients without VA, but long-term mortality was greatly higher.

### Mechanisms of VA Occurrence

Autonomic nerve dysfunction, aberrant sympathetic activation, electrolyte imbalances, and other factors may lead to arrhythmias in patients with severe HF [24, 25]. Although earlier studies reported that LVAD assistance aids in the rehabilitation of cardiac autonomic nerve function in patients with severe HF [26], preoperative dysfunction might still be a source of postoperative VA. At present, the mechanism of VA after LVAD implantation is summarized as primary cardiomyopathy-related, device mechanical stimulation, myocardial scarring, ventricular displacement, electrolyte regulation, and other mechanisms [8].

#### Mechanical Effect

According to the most recent INTERMACS report, CF-LAVD is gradually replacing pulsatile blood flow pumps and has become the first choice for clinical application [27]. Compared to a pulsatile blood flow pump, CF-LAVD is less dependent on preload; however, when preload is significantly reduced or pump rpm is increased, the negative pressure of the inflow tract may aspirate the ventricular septum or the left ventricular free wall into the device, resulting in a “suction event” that leads to myocardial mechanical stimulation and VA [5]. According to Vollkron et al. [28], VA related to a “suction event” occurs suddenly and is relieved with the recovery of preload or the decrease in pump speed.

Furthermore, mechanical stimulation of the device may result in alterations in cardiac structure, changing electrical properties, and aberrant cardiomyocyte repolarization [4]. A prolonged QTc interval usually indicates aberrant cardiomyocyte repolarization. On the first day after LVAD implantation, a longer QTc interval is related to a higher incidence of postoperative VA (RR = 3.3, *p* = 0.01) [17]. The QTc interval increased from 479 at 10 ms to 504 at 11 ms in the first week after LVAD implantation and then decreased to 445 at 11 ms in the second week, according to Harding et al. [29]. Summarizing the results of these two studies, the QTc interval gradually decreases with the extension of LVAD support time, which may explain why VA usually occurs in the early stage after LVAD implantation.

#### Scar-Related Reentrant Arrhythmia

Potential cardiomyopathy after LVAD implantation is frequently accompanied by visible scar formation, which may be the cause of reentrant arrhythmia [30]. A meta-analysis showed that scar-related reentrant arrhythmia is the main mechanism of VT after LVAD implantation [31]. Sutures used in surgical procedures can cause myocardial fibrosis [32]. Pathological studies have shown that stable muscle fibrosis increases the likelihood of arrhythmia by altering cardiomyocyte excitability and increasing ectopic activity [33]. A pathological analysis of the LVAD inflow tract in the myocardium revealed that myocardial fibrosis can progress to apical scar formation, causing VA [34]. Simultaneously, the LVAD inflow channel that penetrates the left ventricular wall forms a special barrier that potentially causes reentrant arrhythmia [9].

#### Electrolyte Regulation

Regulation of the myocardial electrolyte concentration is critical for preserving proper cardiac function. Patients with chronic HF frequently have electrolyte disorders due to changes in neurohumoral regulation and self-regulation as well as the use of diuretic drugs (such as furosemide) and potassium supplement drugs (such as potassium chloride) [35]. However, as tissue and organ perfusion increase following LVAD implantation, various body regulatory systems change, resulting in electrolyte transfer. Ziv et al. [9] retrospectively analyzed the preoperative and postoperative clinical data from 100 patients with LVAD. A multivariate Cox regression analysis showed that abnormal serum electrolyte levels were an independent risk factor for postoperative VA. Monreal and Gerhardt [36] suggested that rapid changes in the electrolyte balance after LVAD implantation increase the risk of postoperative arrhythmias.

#### Others

VA is associated with delayed depolarization of cardiomyocytes, an increase in sodium-calcium exchange, and a change in the calcium concentration [37, 38]. Terracciano et al. [37] discovered that LVAD implantation improves normal calcium homeostasis and reduces abnormal calcium channel regulation in the myocardium of individuals with HF. In addition to the previously mentioned reasons, LVAD implantation may alter the expression of various genes involved in arrhythmia, such as upregulating actin and Ca^2+^ regulatory genes and downregulating connexin 43 and Na^+^/K^+^ ATPase expression [8]. Simultaneously, inotropic drugs used soon after LVAD are proposed to be related to early VA [8].

### Risk Predictors

The risk predictors of VA following LVAD implantation vary depending on the type of LVAD and respondents of the study, similar to epidemiological studies. An understanding of the risk factors or predictors of VA may help doctors diagnose the disease early and intervene when necessary [39]. A preoperative VA history is considered a predictor of VA following LVAD implantation in most studies and, in some cases, is the only predictor of long-term VA after LVAD implantation [19, 23, 40, 41, 42, 43, 44]. Raasch et al. [45] studied 61 LVAD-assisted patients. Only a history of VA prior to implantation was found to be related to the occurrence of VA after LVAD (OR = 13.7, *p* = 0.001). Garan et al. [19] showed that a preoperative VA history (OR = 2.76, *p* = 0.02) and age (OR = 1.04, *p* = 0.03) were predictors of early postoperative VA (within 30 days after operation). Hellman et al. [46] performed a multivariate logistic regression analysis in a retrospective study with a sample size of 85 patients and revealed that a preoperative VA history was not a predictor, and only type B natriuretic peptide level (95% CI: of OR = 1.5–5.1, *p* = 0.0008) was a predictor of postoperative VA in this sample.

Galand et al. [42] collected clinical data from 659 LVAD-assisted patients in 19 medical facilities. Preoperative VA history (OR = 2.32, *p* = 0.001), preoperative atrial fibrillation history (OR = 1.72, *p* = 0.009), idiopathic cardiomyopathy (OR = 1.50, *p* = 0.045), HF duration (>12 months) (OR = 2.58, *p* = 0.001), and no use of angiotensin converting enzyme inhibitors (OR = 2.14, *p* = 0.001) were all found to be risk factors for postoperative VA in the multivariate analysis. Martins et al. [23] also conducted a 652-case multicenter study. In addition to a preoperative VA history (HR = 2.62, *p* = 0.001), the duration of HF (>84 months) (HR = 1.97, *p* = 0.028) was a predictor of postoperative VA.

In addition to the aforementioned risk factors and predictors, the genesis and postoperative management of cardiomyopathy have steadily attracted attention. Ziv et al. [9] and Bedi et al. [21] argue that patients with ischemic cardiomyopathy are more susceptible to developing VA following surgery. Nonischemic cardiomyopathy was reported to be a predictor of early postoperative VA (OR = 2.47, *p* = 0.046) by Garan et al. [19]. However, according to Corre et al. [66], the occurrence of VA following LVAD implantation is unrelated to the type of cardiomyopathy. Refaat et al. [48] analyzed patients with LVAD as the research object in addition to the above risk factors and predictors. VA and nonuse after LVAD implantation were related to the use of receptor blockers (OR = 7.04, *p* = 0.001) [48]. However, a single-center investigation with a sample size of 23 patients found that a lack of use of β-receptor blockers following surgery is not linked to arrhythmia [18].

### Surveillance and Treatment

Multiple mechanisms and factors should be considered for the prevention and treatment of postoperative VA in patients with long-term VAD assistance. Pharmacotherapy is a key link in the management of postoperative VA according to the causes and risk factors discussed in the preceding sections, in addition to the preventative and therapeutic measures described above. β-Receptor blockers have been shown to exert a strong antiarrhythmic effect [49]. Although information on VAD from randomized and prospective studies on the use of receptor blockers is currently lacking, receptor blockers may be used in patients without significant contraindications, such as right ventricular dysfunction, based on a comprehensive consideration of individualized therapy. In addition to using routine medication (e.g., receptor blockers, amiodarone, lidocaine, etc.), clinicians should pay close attention to the changes in electrolyte levels, correct electrolyte disorders, and comprehensively analyze changes in hemodynamics and the causes of VA. Effective monitoring or etiological treatment should be implemented.

#### Prevention of “Suction Events”

“Suction events” have been identified as a common cause of VA following LVAD implantation. During monitoring and treatment, “suction events” are frequently characterized by a reduced flow, a characteristic flow profile, and increased power of the blood pump operation, among others, and should be noted and corrected as soon as possible. Body position may affect the spatial positional relationship of the inflow tract with the interventricular septum and ventricular free wall. Thus, real-time assessments of left ventricular geometry using perioperative echocardiography are also critical to avoid postoperative VA due to mechanical stimulation [50]. Adjusting the pump speed once a “suction event” occurs helps to restore the left ventricular volume status and reduces the likelihood of VA occurrence [51]. If changes in pump speed or volume status are insufficient to correct VA caused by contact between the VAD inflow tract and the myocardium, reoperation with adjustments to the access tract position should be considered [5].

#### Ablation

After a thorough assessment of the patient's situation, treatments such as ablation should be performed before VAD implantation in patients with a history of preoperative VA to increase the patient's survival rate after VAD implantation [52]. If the patient's preoperative conditions do not allow ablation prior to implantation, surgical ablation during the same period of VAD implantation should be considered [53]. VA ablation will be limited after LVAD implantation. On the one hand, a pericardial adhesion is generated after VAD implantation, essentially resulting in a limitation on epicardial ablation; on the other hand, the VAD cannula limits the use of intracardiac ablation. However, for patients with medically uncontrolled VA after LVAD implantation, intracardiac ablation remains an option. Among a sample size of 34 patients who experienced VA following HeartMate II implantation, 13 were free of recurrent arrhythmias after 25 ± 15 months of follow-up [51]. Another study involved 21 patients (15 HeartMate II®, 6 HeartWare®) and found that 64% of patients were free of recurrent VA after ablation, with 1-year survival rates of 67% for nonrecurrent patients and 29% for recurrent patients (*p* = 0.049) [54].

#### Implanted Cardioverter Defibrillators

Implantable cardioverter defibrillators (ICDs) are currently considered primary and secondary prevention techniques for sudden cardiac death in patients with HF, but perspectives on their utility in patients following VAD implantation are mixed [55]. A case of electromagnetic interference produced by ICD and LVAD was described by Moini et al. [56]. Erqou et al. [47] described a case in which the LVAD interfered with the ICD function due to electromagnetic interference. According to previous studies, an interaction between LVAD and ICD devices is uncommon, with only 2 of 76 patients (HeartMate II®) experiencing such an interaction [57]. Yalcin et al. [58] performed a single-center retrospective study to analyze the clinical data from 86 patients with LVAD and ICD implantation (46 patients implanted with HeartMate II® and 40 patients implanted with HeartMate III®). The incidence of electromagnetic interference in patients implanted with the HeartMate II® and ICD or HeartMate III® and ICD was 15% and 11%, respectively.

#### Others

By decreasing sympathetic activity, sympathetic denervation has been shown to diminish the occurrence of persistent VA or the usage of ICD [59]. Vlismas et al. [60] reported that sympathetic denervation was performed in 1 female patient with intermittent VA after LVAD implantation, and no recurrence was observed at the 8-month postoperative follow-up, implying that sympathetic denervation may be considered for patients with postoperative VA implanted with LVADs who are refractory to medical, ablative, or ICD therapy.

## Conclusion and Outlook

With the widespread use of LVAD in clinical practice, postoperative VA has become a common clinical complication. Although postoperative VA with LVAD has no effect on short-term hemodynamics in many individual patients, its long-term persistence or recurrence still poses risks of increased postoperative mortality and rehospitalization. By summarizing case data from various centers, we discovered that even if severe VA (such as VF) occurs, patients still have the possibility of maintaining hemodynamic stability with the support of LVAD; however, the ability to maintain circulation remains unclear. Our study revealed that before LVAD is implanted, the burden of arrhythmias should be considered and thoroughly assessed because the prior VA is the strongest predictor for post-LVAD VA. Even though post-LVAD VA occurs commonly in post-LVAD patients, it did not significantly increase the mortality. Ablation has shown promise as a routine strategy to prevent or cure postoperative VA by preventing “suction events.” The therapeutic efficacy and long-term effects of ICD and sympathetic denervation are not fully understood.

In the future, relevant acute and chronic animal experiments should be conducted to examine the effect of the postoperative VA duration on vital signs and circulatory conditions, as well as the reasons for short-term hemodynamic stability during VF. New management strategies that contain patient baseline features, prior history of VA, proper scheduling of ablation therapy, and effective medical treatment will certainly help us better understand VA occurrence after LVAD implantation and the final endings in LVAD patients. Future multicenter prospective studies with uniform patient follow-up methods are needed to screen additional potential risk factors and predictors. This approach will help define the incidence rate of VA after LAVD implantation. As a result, we will be able to guide the selection of preventive interventions.

## Limitations

The majority of current relevant studies are single-center, small-sample, and retrospective, and the risk factors and predictors examined may be insufficient and vary widely and additional multicenter prospective studies are needed. Another limitation is the inclusion of studies that examined CF-LVAD patients who specifically received ablation for the treatment of VA. This may have raised the rate of ablation in our population inadvertently.

## Statement of Ethics

An ethics statement was not required for this study type, no human or animal subjects or materials were used.

## Conflict of Interest Statement

The authors have no conflicts of interest to declare.

## Funding Sources

This study was supported by grants from the National Key Research and Development Program of China (Project number: 2017YFC0111005) and Natural Science Foundation of Tianjin, China (Project number: 18JCZDJC36200).

## Author Contributions

Jianwei Shi: conception/design, design, data collection, and drafting the article. Xinyi Yu: data collection and data interpretation. Zhigang Liu: conception/design, data interpretation, critical revision of the article, and approval of the article.

## Data Availability Statement

The datasets used and/or analyzed during the present study are available from the corresponding author upon reasonable request.

## Figures and Tables

**Table 1:** Characteristics of studies included [18, 21, 40, 45, 51, 54, 61, 62, 63, 64, 65, 67]

Author	Year published	Journal	Study date	Study institution	Type of study	LVAD patients, *n*	VA patients, *n*	Type of LVAD	Key findings
Andersen et al. [18]	2009	J Heart Lung Transplant	March 2006 to July 2008	The Heart Centre, Rigshospitalet, Copenhagen, Denmark	Retrospective	23	12	HMII (Thoratec, Pleasanton, CA, USA)	About 50% of patients suffered at least an episode of VA after CF-LVAD implantation and no clear predictors be defined. A quarter of patients had significant hemodynamic instability with VA occurred which led the authors to advise to consider performing a prophylactic ICD in CF-LVAD patients

Bedi et al. [21]	2007	Am J Cardiol	January 1987 to June 2001	University of Pittsburgh Medical Center, PA, USA	Retrospective	111	24	Unknown	LVAD patients with ischemic HF history are more frequent occurring VA, and their occurrence is associated with greater mortality

Boudghène-Stambouli et al. [61]	2014	J Interv Card Electrophysiol	June 2007 to August 2012	Lille University Hospital, Lille, France	Retrospective	26	11	HMII (Thoratec, Pleasanton, CA, USA)	About 50% of CF-LVAD implantation patients experienced VA, and prior VA is the strongest predictor

Efimova et al. [62]	2017	Heart Rhythm	May 2011 to December 2013	Heart Center Leipzig, Leipzig, Germany	Prospectively	98	48	HMII (Thoratec, Pleasanton, CA, USA) or Heartware (Heartware, Sydney, NSW, Australia)	Pre-LVAD VA and atrial fibrillation were predictive of VA after CF-LVAD. The occurrence of VA in LVAD implantation patients is greatly high

Enriquez et al. [63]	2013	Circ Arrhythm Electrophysiol	June 2008 to April 2012	Mount Sinai Medical Center, NY, USA	Retrospective	106	37	HMII (Thoratec, Pleasanton, CA, USA)	VA occurs commonly in CF-LVAD patients but VAs are not associated with a worse prognosis, and simultaneous use ICDs may not provide a survival benefit

Greet et al. [67]	2018	JACC Clin Electrophysiol	January 2000 to April 2015	Baylor College of Medicine Houston, TX, USA	Retrospective	517	133	HMII (Thoratec, Pleasanton, CA) or Heartware (Heartware, Sydney, Australia) or other CFLVAD	VA occur in 30 days after CF-LVAD implantation are related to increased mortality. Prior cardiac surgery and VT storm are predictors of early VA

Kumar et al. [65]	2019	J Interv Card Electrophysiol	January 2008 to March 2018	Hartford Hospital, Hartford, CT, USA	Retrospective	157	48	HMII II (Thoratec, Pleasanton, CA, USA) or Heartware (Heartware, Sydney, NSW, Australia)	VA is common in CF-LVAD patients and pre-LVAD VA is a predictor of post-LVAD VA. Neither VA nor ICD shocks are correlated with mortality

Moss et al. [54]	2017	JACC Clin Electrophysiol	July 2010 to April 2016	University of Chicago Medicine, Chicago, IL, USA	Retrospective	21	21	HMII (Thoratec, Pleasanton, CA, USA) or Heartware (Heartware, Sydney, NSW, Australia)	Ablation as destination therapy in post-LVAD patients and freedom from recurrent VT and ICD shocks were related to improved 1-year survival

Oswald et al. [40]	2010	Eur J Heart Fail	July 2005 to October 2008	Hannover Medical School, Hannover, Germany	Prospective	61	21	HMII II (Thoratec, Pleasanton, CA, USA) or Heartware (Heartware, Sydney, NSW, Australia)	ICD therapy is safe and effective in LVAD patients. Prior VA greatly predicts post-LVAD VA and further use of ICD

Raasch et al. [45]	2012	Am Heart J	January 2006 to February 2011	University of North Carolina, Chapel Hill, NC, USA	Retrospective	61	26	HMII (Thoratec Corp, Pleasanton, CA) or Jarvik2000 (Jarvik Heart Inc, New York, NY, USA)	VA occurs commonly in LVAD patients but did not significantly impact survival or transplantation rates
Sacher et al. [51]	2015	Circ Arrhythm Electrophysiol	2009–2014	Bordeaux University, Bordeaux, France Brigham and Women Hospital, Boston, MA, USA Hospital of the University of Pennsylvania, Philadelphia, PA, USA University of Wisconsin Hospital, Madison, Wl, USA University of Miami Medical Center, Miami, FL, USA Institute for Clinical and Experimental Medicine, Prague, Czech Republic Medical College of Virginia/Virginia Commonwealth University School of Medicine, Richmond, VA, USA Richmond CHU de Toulouse, Toulouse, France Mount Sinai Hospital, New York, NY, USA	Retrospective	34	34	HMII (Thoratec, Pleasanton, CA, USA)	All patients who occur VT after LVAD implantation have a history of VT before LVAD implantation. Pre-LVAD VA predict post-LVAD VA

Yoruk et al. [64]	2016	Heart Rhythm	January 2005 to September 2013	University of Rochester Medical Center, Rochester, NY, USA	Retrospective	149	41	HMII (Thoratec, Pleasanton, CA, USA)	History of VA and AF strongly predict post-LVAD VA which is associated with an increased risk of all-cause mortality

**Table 2:** Baseline characteristics of patients who experienced VA after LVAD implantation

	Pooled value [95% CI]	Studies, *n*	Patients, *n* (*N* or *n/N*)	*I*^2^, %
Male, %	83 [0.77, 0.89]	10	322/398	60
Past medical history, %				
Diabetes mellitus	40 [0.32, 0.49]	4	105/248	40
Hypertension	58 [0.52, 0.64]	4	144/248	0
HF etiology, %				
Ischemic	53 [0.44, 0.62]	10	156/302	60
Nonischemic	47 [0.38, 0.56]	10	146/302	60
Pump type, %				
HeartMate II LVAD	85 [0.78, 0.93]	9	300/390	95
HeartWare HVAD	4 [.01, 0.06]	9	28/390	69
Other CF-LVAD	3 [0.00, 0.05]	11	35/432	76
Indication for CF-LVAD, %				
Bridge to transplant	58 [0.45, 0.70]	5	170/285	77
Destination therapy	42 [0.29, 0.54]	5	113/285	76

**Table 3:** Characteristics of VA after LVAD implantation

	Pooled value [95% CI]	Studies, *n*	Patients, *n* (*N* or *n/N)*	*I*^2^,%
History of arrhythmias, %				
Atrial fibrillation	53 [0.25, 0.81]	4	118/311	96
VA	61 [0.52, 0.69]	9	164/278	59
New VA after LVAD, %	39 [0.29, 0.49]	8	93/230	64
VA ≤30 days, %	59 [0.51, 0.67]	7	177/311	43
Treatment for VA, %				
Anti-tachycardial pacing	27 [0.04, 0.49]	3	12/49	69
Cardioversion	69 [0.57, 0.82]	3	34/49	0
Ablation	74 [0.47, 1.01]	3	65/103	99

**Table 4:** Outcomes of CF-LVAD patients who experienced VA after implantation

	Pooled value [95% CI]	Studies, *n*	Patients, *n* (*N* or n/N)	*I*^2^, %
Mortality, 30 days, %	4 [0.01, 0.07]	6	17/292	55
Mortality, overall, %	28 [0.15, 0.41]	5	67/255	80
Transplanted, %	25 [0.13, 0.36]	2	14/55	5
